# A case-control study of risk factors for fetal and early neonatal deaths in a tertiary hospital in Kenya

**DOI:** 10.1186/s12884-014-0389-8

**Published:** 2014-11-29

**Authors:** Faith Yego, Catherine D’Este, Julie Byles, Paul Nyongesa, Jennifer Stewart Williams

**Affiliations:** Department of Health Policy and Management, Moi University, College of Health Sciences, Eldoret, 30100 Kenya; Chair in Biostatistics, National Centre for Epidemiology and Population Health, Research School of Population Health, The ANU College of Medicine Biology and Environment, the Australian National University, Canberra, ACT Australia; Research Centre for Gender, Health and Ageing, University of Newcastle, Hunter Medical Research Institute, New Lambton Heights, NSW Australia; Department of Reproductive Health, Moi University, College of Health Sciences, Eldoret, 30100 Kenya; Conjoint Academic, Research Centre for Gender, Health and Ageing, University of Newcastle, Newcastle, Australia; Research Engineer, Umeå Centre for Global Health Research, Unit of Epidemiology and Global Health, Department of Public Health and Clinical Medicine, Faculty of Medicine, Umeå University, Umeå, Sweden

**Keywords:** Early neonatal mortality, Fetal death, Risk factors, Maternal, Tertiary hospital

## Abstract

**Background:**

It is important to understand the risk factors for fetal and neonatal mortality which is a major contributor to high under five deaths globally. Fetal and neonatal mortality is a sensitive indicator of maternal health in society. This study aimed to examine the risk factors for fetal and early neonatal mortality at the Moi Teaching and Referral Hospital in Kenya.

**Methods:**

This was a case-control study. Cases were fetal and early neonatal deaths (n = 200). The controls were infants born alive immediately preceding and following the cases (n = 400). Bivariate comparisons and multiple logistic regression analyses were undertaken.

**Results:**

The odds of having 0-1 antenatal visits relative to 2-3 visits were higher for cases than controls (Adjusted Odds Ratio (AOR) = 4.5; 95% CI: 1.2-16.7; p = 0.03)). There were lower odds among cases of having a doctor rather than a midwife as a birth attendant (AOR = 0.2; 95% CI: 0.1-0.6; p < 0.01). The odds of mothers having Premature Rupture of Membranes (AOR = 4.1; 95% CI: 1.4-12.1; p = 0.01), haemorrhage (AOR = 4.8; 95% CI: 1.1-21.9; p = 0.04) and dystocia (AOR = 3.6; 95% CI: 1.2-10.9; p = 0.02) were higher for the cases compared with the controls. The odds of gestational age less than 37 weeks (AOR = 7.0; 95% CI 2.4-20.4) and above 42 weeks (AOR = 16.2; 95% CI 2.8-92.3) compared to 37-42 weeks, were higher for cases relative to controls (p < 0.01). Cases had higher odds of being born with congenital malformations (AOR = 6.3; 95% CI: 1.2-31.6; p = 0.04) and with Apgar scores of below six at five minutes (AOR = 26.4; 95% CI: 6.1-113.8; p < 0.001).

**Conclusion:**

Interventions that focus on educating mothers on antenatal attendance, screening, monitoring and management of maternal conditions during the antenatal period should be strengthened. Doctor attendance at each birth and for emergency admissions is important to ensure early neonatal survival and avert potential risk factors for mortality.

## Background

Neonatal death is defined as newborn death occurring within the first four weeks after birth [1]. Neonatal deaths represent 40% of under-five deaths worldwide [2,3]. Despite the reduction in overall under five mortality in developing regions, from 97 deaths per 1000 live births in 1990 to 63 per 1000 live births in 2010, there has been little change in neonatal deaths as a proportion of under five deaths [2,3]. In Sub-Saharan Africa, for example, the proportion of neonatal deaths among under five deaths increased from 37% in 1990 to 40% in 2010 [2,3]. Neonatal mortality rates in the developing world are generally high, for example, there were 32 deaths per 1000 live births in Central Africa in 2009 [4], and in Somalia the estimated neonatal mortality rate was 52 per 1000 live births in 2012 [5]. In comparison, the neonatal mortality rate in Kenya is currently 28 per 1000 live births and there has been little progress towards achieving the Millennium Development Goal (MDG) Four for child survival [2,6]. The main direct causes of neonatal deaths globally are preterm births (27%), severe infections (26%), asphyxia (23%), and neonatal tetanus (7%) [3,7,8].

Early neonatal death is defined as all deaths of live-born infants occurring on or before the first seven days of life [9]. There is evidence that there has been no measurable reduction in early neonatal mortality over the past decade [2]. Most programs addressing childhood mortality focus on pneumonia, malaria, diarrhoea and vaccine preventable conditions, which are geared towards improving child survival after the first four weeks of life [5,6]. There is evidence that the highest numbers of early neonatal deaths in Sub-Saharan Africa are due to infections [10,11].

Fetal death is defined as any fetus born without a heartbeat, respiratory effort or movement, or any other sign of life [9]. It is estimated that globally 2.9 million babies experience fetal death or die within the first week of life, with 99% of these deaths occurring in developing countries [12]. Studies have shown that 50% of maternal deaths occur within the first day after childbirth and approximately 30% of stillbirths occur during labour [5].

The health and survival of newborns has been shown to be closely linked to that of their mothers, since inadequate maternal care during the pregnancy and postpartum period can also affect the neonate [13]. It has been suggested that access to antenatal care and emergency obstetric care could reduce neonatal mortality by 10-15% [5,14]. There is evidence that 10% of intrapartum-related and preterm deaths can be reduced by immediate assessment and stimulation of newborns [15]. Treatment with antenatal corticosteroids has been associated with a decrease in overall neonatal deaths, especially for women with premature rupture of membranes (PROM) [16].

Health system factors have been associated with newborn deaths, especially in low resource settings where quality of care is generally poor and inadequate [17]. An assessment of six African countries showed that less than 12% of personnel working in health facilities were trained to conduct neonatal resuscitation and no more than 22% of the facilities had sufficient equipment for neonatal resuscitation [18].

Due to these high mortality rates it is important to understand the risk factors for fetal and neonatal mortality which are major contributors to high under five deaths globally. Fetal and neonatal mortality is also a sensitive indicator of maternal health in society because healthy mothers give birth to healthy babies. This study was undertaken to assess maternal and neonatal risk factors associated with fetal and early neonatal deaths in the second largest tertiary hospital in Kenya in order to provide insights into the circumstances surrounding fetal and early neonatal deaths.

## Methods

This retrospective case-control study was conducted in Moi Teaching Referral Hospital (MTRH) in Kenya. The study was undertaken in Uasin Gishu County which is in the Rift Valley Province of Kenya. This hospital services approximately seven million women [19]. The MTRH has a reproductive health wing called the Riley Mother Baby Unit, which contains both the labour ward and the New Born Unit (NBU). The NBU has three sections: the born before arrival (BBA) unit, the acute ward or critical ward and the general ward [20]. The NBU has several incubators, newborn trolleys, a capacity of 60 beds, and is staffed with consultant paediatricians (10), registrars (6), intern doctors (one) and nurses (30) specially trained for NBU care [20]. According to the MTRH records department the total number of monthly admissions during the study period was about 120-140 newborns [20].

Client flow in the hospital is such that pregnant/post-partum patients are seen in a designated room (triage area) on the labour ward floor by a nurse, medical officer intern and resident doctor. Patients in active labour are usually admitted to the labour ward where they are managed by a team of obstetricians, residents, interns and midwives/nurses. Patients in a latent phase of labour, or with medical conditions, are usually admitted to the antenatal ward, and other patients with medical issues after delivery are admitted to the post natal ward. Patients without complications are discharged. Neonates who have complications after birth are immediately taken to the NBU which is adjacent to the labour ward. The neonatologist and paediatricians subsequently manage the neonate [20].

A medical record review was undertaken on admissions to the newborn unit in MTRH between January 2005 and March 2011. Cases were defined as neonates who were born dead (fetal deaths) or died within seven days of birth (early neonatal deaths). Most recent cases were selected retrospectively until the desired sample size was achieved. Two controls were obtained for every case. The controls were surviving neonates born immediately preceding and following the cases within the first week of life. Exclusion criteria were late neonatal deaths (more than seven days after birth).

A structured data collection instrument was used to collect data from medical records identified from the NBU admission register. It was not possible to blind the data abstractors to case/control status because mortality information was in the medical records.

The primary cause of death was identified using the information from hospital medical records and post mortem reports. Where interpretation was required, the information on the cause of death was verified by the study physician. Data were coded and double entered into two separate password protected databases, which were later compared for consistency. Where there were inconsistencies, the patient’s file was obtained to verify the information.

The outcome of this study was death of the neonate or fetus at birth or within seven days of birth. Explanatory variables were classified as maternal and obstetric characteristics, maternal and obstetric complications, neonatal complications and neonatal characteristics.

*Maternal and obstetric characteristics* included information on women of reproductive age (15 to 49 years) such as: mother’s age (15-24 years, 25-34 years, or 35-45 years); gravidity categorised as primigravida (1), multigravida (2-4), or grandmultigravida (above 5); qualification of birth attendant (doctor (consultant/registrar/intern), or midwife); gestational age (less than 37 weeks, 37-42 weeks or above 42 weeks); mode of delivery (spontaneous vertex delivery, assisted vaginal delivery, or caesarean section); number of antenatal visits (ANC) (0-1, 2-3, or above 4), and booking status on admission (yes = attended ANC at MTRH or no = did not attend ANC at MTRH).

*Maternal and obstetric complications* included the presence of common causes of death, as documented in the patient records and post mortem reports; premature rupture of membranes (PROM); dystocia (prolonged or obstructed labour); pre-eclampsia; haemorrhage; and other complications (cardio respiratory diseases, previous scar, Human Immunodeficiency Virus or HIV, malaria, retained placenta, anaemia, abortion). A complication was assumed there was an indication to this effect in the notes, otherwise it was assumed that there were no complications.

*Newborn complications* included the presence of causes of newborn death as documented in the records including asphyxia, congenital malformation, sepsis, Respiratory Distress Syndrome (RDS), and other complications (hypothermia, diarrhoea, jaundice, hypoglycaemia, meconium aspiration syndrome, and HIV). One single cause of death was identified for each neonate case and where there were multiple causes the final cause of death documented in the post mortem reports was used.

*Neonatal characteristics* including sex, Apgar score at five minutes and weight in grams were also recorded.

Ethical approval was provided by the University of Newcastle Human Research Ethics Committee (HREC) and the Institutional Research and Ethics Committee (IREC) in Kenya. Permission was obtained from the MTRH administration to undertake the study.

### Statistical methods

Statistical analysis was performed using STATA version 10 (StataCorp, College Station, TX, USA). Exploratory data analysis involved checking the data for implausible relationships, outliers and errors using frequency distributions, tables and graphs. Checks comprised visualizing the distributions by use of graphics including histograms overlaid with a normal curve, normal probability plots and box plots to identify potential errors. Bar charts were also used to compare distributions between groups.

All variables were categorical. Categories were combined where cell sizes were small. Bivariate analysis was undertaken using the Chi-squared test to compare characteristics of cases and controls. Initially, a modelling process was undertaken by including all variables with p < 0.2 in separate models for each of the four groups of potential risk factors (maternal and obstetric characteristics, neonatal complications, maternal and obstetric complications and neonatal characteristics). Each group of potential risk factors was analysed in separate multivariable logistic regression. A backward stepwise method was used whereby, at each step, variables with a p-value of >0.1 on the likelihood ratio test were removed. The remaining variables were combined in a final model. Unadjusted and adjusted odds ratios, confidence intervals and p-values are reported for all models.

For a ratio of cases to controls of 1:2, 80% power, a 5% significance level and 40% probability of exposure (i.e. risk factor prevalence) in controls, a sample of 600 neonates (200 cases and 400 controls) was required to detect an absolute difference in risk factor prevalence of at least 12%, or an odds ratio of approximately 0.6 or 1.7.

## Results

A total of 600 records were reviewed (200 cases and 400 controls) from January 2005 to March 2011. As with many studies using data abstracted from medical records, data were incomplete in some areas. The proportion of missing data in variables ranged from 0.3% to 22%. Data were separately stored in the MTRH neonatal and maternal records. Missing data in individual records arose from one or both sources.

Table 1 shows the maternal and obstetric factors associated with fetal and early neonatal mortality at MTRH. Gestational age at admission (p < 0.001), number of antenatal visits (p < 0.001) and qualification of birth attendant (p = 0.01) were all significantly associated with fetal and early neonatal mortality. The odds of gestational period below 37 weeks, relative to gestational age of 37-42 weeks were higher for cases than controls (Adjusted Odds Ratio (AOR) = 16.6; 95% CI: 8.2-33.7). The odds of having 0-1 antenatal visits relative to 2-3 visits were higher for cases than controls (AOR = 5.4; 95% CI: 2.0-14.7). Compared to controls, cases had lower odds of having four or more antenatal visits relative to 2-3 visits (AOR = 0.3; 95% CI: 0.1-0.7); and having a birth attendant who was a doctor rather than a midwife (AOR = 0.4; 95% CI: 0.2-0.8).

**Table 1:** **Association between maternal and obstetric factors with fetal and early neonatal death at MTRH from Jan 2005-Mar 2011**

**Predictor**	**Cases n (%)**	**Controls n (%)**	**Unadjusted OR (95% CI)**	**Adjusted OR (95% CI)**	**p-value for likelihood ratio test**
**Age in years**					0.98
15-24	97 (51)	195 (49)	1.0	1.0	
25-34	68 (35)	172 (43)	0.8 (0.5-1.2)	1.1 (0.5-2.3)	
35-45	27 (14)	33 (8)	1.6 (0.9-2.9)	1.1 (0.3-4.0)	
**Gravidity**					0.26
Multigravida	108 (54)	231 (58)	1.0	1.0	
Primigravida	58 (29)	119 (30)	1.0 (0.7-1.5)	0.8 (0.4-1.9)	
Grandmultigravida	34 (17)	50 (12)	1.5 (0.9-2.4)	2.3 (0.8-6.6)	
**Qualification of birth attendant**					0.01
Midwife	142 (74)	191 (50)	1.0	1.0	
Doctor	50 (26)	187 (49)	0.4 (0.2-0.5)	0.4 (0.2-0.8)	
**Gestational age at admission**					<0.001
<37 weeks	116 (72)	74 (20)	11.0 (7.1-17.2)	16.6 (8.2-33.7)	
37-42 weeks	39 (24)	274 (75)	1.0	1.0	
> 42 weeks	6 (4)	15 (4)	2.8 (1.0-7.7)	2.4 (0.4-13.4)	
**Mode of delivery**					0.79
Normal	146 (73)	249 (62)	1.0	1.0	
Assisted	9 (5)	13 (3)	1.2 (0.5-2.8)	1.5 (0.3-7.9)	
	45 (22)	138 (35)	0.6 (0.4-0.8)	1.3 (0.5-3.1)	
**Number of antenatal care visits**					<0.001
0-1	31 (26)	15 (4)	5.9 (3.0-11.6)	5.4 (2.0-14.7)	
2-3	74 (63)	212 (59)	1.01	1.0	
Above 4	13 (11)	133 (36)	0.3 (0.1-0.5)	0.3 (0.1-0.7)	
**Booking status**					0.28
No	139 (79)	311 (80)	1.0	1.0	
Yes	38 (21)	80 (20)	0.9 (0.6-1.5)	0.9 (0.5-2.2)	

Reference category represented by 1.0.

Numbers may not add to total sample size due to missing values.

Maternal obstetric complications associated with fetal and early neonatal mortality at MTRH are shown in Table 2. PROM, haemorrhage, and dystocia were significantly associated with mortality. Compared with the controls, the cases had higher odds of maternal PROM (AOR = 5.9; 95% CI: 3.5-9.9; p < 0.001), dystocia (AOR = 1.9; 95% CI: 1.2-3.1; p = 0.01), and haemorrhage (AOR = 2.4; 95% CI: 1.2-4.7; p = 0.02). Cases had higher odds of other complications compared with controls (AOR =2.0; 95% CI: 1.0-3.9; p = 0.06) although the difference was not significant.

**Table 2:** **Association between maternal obstetric complications with fetal and early neonatal mortality at MTRH from Jan 2005-Mar 2011**

**Predictor**	**Cases n (%)**	**Controls n (%)**	**Unadjusted OR (95% CI)**	**Adjusted OR (95% CI)**	**p-value for likelihood ratio test**
**Premature rupture of membranes**					<0.001
Yes	52 (26)	27 (7)	4.9 (2.9-8.0)	5.9 (3.5-9.9)	
No	148 (74)	373 (93)	1.0	1.0	
**Dystocia**					0.01
Yes	42 (21)	64 (16)	1.4 (0.9-2.2)	1.9 (1.2-3.1)	
No	158 (79)	336 (84)	1.0	1.0	
**Pre-eclamspia**					0.44
Yes	13 (7)	29 (7)	1.0 (0.5-1.8)	1.3 (0.7-2.7)	
No	187 (93)	371 (93)	1.0	1.0	
**Haemorrhage**					0.02
Yes	17 (9)	21 (5)	1.7 (0.9-3.3)	2.4 (1.2-4.7)	
No	183 (91)	379 (95)	1.0	1.0	
**Other complications**					0.06
Yes	16 (8)	22 (6)	1.5 (0.8-2.9)	2.0 (1.0-3.9)	
No	184 (92)	378 (94)	1.0	1.0	

Reference category for logistic regression represented by 1.0.

Other complications included: cardio respiratory diseases, previous scar, HIV, malaria, retained placenta, anaemia, abortion.

Numbers may not add to total sample size due to missing values.

Table 3 shows the association between neonatal complications with fetal and early neonatal mortality at MTRH. The odds of asphyxia (AOR 2.4; 95% CI: 1.6-3.6; p < 0.001), congenital malformation (AOR 2.9; 95% CI: 1.5-5.7; p = 0.01) and RDS (AOR 1.6; 95% CI: 1.1-2.4; p = 0.01), were higher for cases relative to controls. The odds of sepsis were marginally non-significantly lower for cases than controls (AOR = 0.7; 95% CI: 0.4-1.0; p = 0.06).

**Table 3:** **Association between neonatal complications with fetal and early neonatal mortality at MTRH from Jan 2005-Mar 2011**

**Predictor**	**Cases n (%)**	**Controls n (%)**	**Unadjusted OR (95% CI)**	**Adjusted OR (95% CI)**	**p-value for likelihood ratio test**
**Asphyxia**					<0.001
Yes	73 (37)	74 (19)	2.5 (1.7-3.7)	2.4 (1.6-3.6)	
No	127 (63)	326 (81)	1.0	1.0	
**Congenital malformation**					0.01
Yes	21 (11)	18 (5)	2.5 (1.3-4.8)	2.9 (1.5-5.7)	
No	179 (89)	182 (95)	1.0	1.0	
**Sepsis**					0.06
Yes	37 (18)	125 (31)	0.5 (0.3-0.8)	0.7 (0.4-1.0)	
No	163 (82)	275 (69)	1.0	1.0	
**Respiratory Distress syndrome**					0.01
Yes	66 (33)	94 (24)	1.6 (1.1-2.3)	1.6 (1.1-2.4)	
No	134 (67)	306 (76)	1.0	1.0	
**Other neonatal complications**					0.50
Yes	32 (16)	79 (20)	0.8 (0.5-1.2)	0.9 (0.5-1.4)	
No	168 (84)	321 (80)	1.0	1.0	

Reference category for logistic regression represented by 1.0.

Other complications included: hypothermia, diarrhoea, jaundice, hypoglycaemia, meconium aspiration syndrome, sero-exposed.

Numbers may not add to total sample size due to missing values.

The association between neonatal characteristics and early neonatal mortality at MTRH is shown in Table 4. Baby’s birth weight and Apgar score were significantly associated with mortality (p < 0.001 for both). Relative to controls, cases had higher odds of birth weight less than 2500 grams (AOR 6.6; 95% CI: 3.8-10.2) and an Apgar score of zero to six (AOR 13.4; 95% CI 7.3-24.8) rather than seven or above at five minutes.

**Table 4:** **Association between neonatal characteristics with fetal and early neonatal mortality at MTRH from Jan 2005-Mar 2011**

**Predictor**	**Cases n (%)**	**Controls n (%)**	**Unadjusted OR (95% CI)**	**Adjusted OR (95% CI)**	**p-value for likelihood ratio test**
Baby’s birth weight					<0.001
less than 2500 gms	133 (73)	116 (29)	6.6 (4.5-9.8)	6.6 (3.8-10.2)	
Above 2500 gms	49 (27)	282 (71)	1.0	1.0	
Baby’s Apgar score					<0.001
0-6 at 5 mins	78 (49)	20 (5)	17.2 (10.0-29.8)	13.4 (7.3-24.8)	
7-10 at 5 mins	79 (50)	349 (95)	1.0	1.0	
Baby’s sex					
Female	85 (44)	190 (48)	0.9 (0.6-1.3)	1.0 (0.6-1.7)	0.88
Male	105 (55)	208 (52)	1.0	1.0	

Reference category for logistic regression represented by 1.0.

Numbers may not add to total sample size due to missing values.

Table 5 represents the final model combining factors from the four previous models. The odds of having a birth attendant who was a doctor versus a midwife were lower for cases relative to controls (AOR = 0.2; 95% CI: 0.1-0.6; p < 0.01). Cases, compared to controls, had higher odds of having mothers who had 0-1 antenatal visit relative to 2-3 visits (AOR = 4.5; 95% CI: 1.2-16.7; p = 0.03 overall). The odds of gestational age less than 37 weeks (AOR = 7.0; 95% CI 2.4-20.4) and above 42 weeks (AOR = 16.2; 95% CI 2.8-92.3), rather than 37-42 weeks were higher for cases relative to controls (p < 0.01). The odds of mothers with complications of PROM (AOR = 4.1; 95% CI: 1.4-12.1; p = 0.01), haemorrhage (AOR = 4.8; 95% CI: 1.1-21.9; p = 0.04) or dystocia (AOR = 3.6; 95% CI: 1.2-10.9; p = 0.02) were higher for cases relative to controls. Cases, compared to controls had higher odds of being born with congenital malformations (AOR = 6.3; 95% CI: 1.2-31.6; p = 0.04), and being born with Apgar scores of 0-6 (AOR = 26.4; 95% CI: 6.1-113.8; p < 0.001), rather than a score of above seven at five minutes.

**Table 5:** **Determinants of fetal and early neonatal mortality at MTRH from Jan 2005-Mar 2011**

				**LR test statistic**		
**Predictor**	**Cases n (%)**	**Controls n (%)**	**Adjusted OR (95% CI)**	**x** ^**2**^	**Degrees of freedom**	**P value**
Qualification of birth attendant				10.58	1	<0.01
Midwife	142 (74)	191 (50)	1			
Doctor	50 (26)	187 (49)	0.2 (0.1-0.6)			
Number of antenatal care visits				7.35	2	0.03
0-1	31 (26)	15 (4)	4.5 (1.2-16.7)			
2-3	74 (63)	212 (59)	1.0			
Above 4	13 (11)	133 (36)	0.5 (0.2-1.5)			
Gestational age at admission				19.72	2	<0.01
<37 weeks	116 (72)	74 (20)	7.0 (2.4-20.4)			
37-42 weeks	39 (24)	274 (75)	1.0			
> 42 weeks	6 (4)	15 (4)	16.2 (2.8-92.3)			
Premature rupture of membrane				6.38	1	0.01
No	148 (74)	373 (93)	1.0			
Yes	52 (26)	27 (7)	4.1 (1.4-12.1)			
Haemorrhage				4.09	1	0.04
No	183 (91)	371 (95)	1.0			
Yes	17 (9)	29 (5)	4.8 (1.1-21.9)			
Dystocia				5.08	1	0.02
No	158 (79)	336 (84)	1.0			
Yes	42 (21)	64 (16)	3.6 (1.2-10.9)			
Other maternal complications				0.47	1	0.49
No	184 (92)	378 (94)	2.0 (0.3-13.9)			
Yes	16 (8)	22 (6)	1.0			
Sepsis				0.3	1	0.58
No	163 (82)	275 (69)	1.0			
Yes	37 (18)	125 (31)	1.4 (0.5-3.9)			
Asphyxia				1.65	1	0.20
No	127 (63)	326 (81)	1.0			
Yes	73 (37)	74 (19)	0.4 (0.1-1.7)			
Respiratory Distress syndrome				0.05	1	0.82
No	134 (67)	306 (76)	1.0			
Yes	66 (33)	94 (24)	0.9 (0.4-2.3)			
Congenital malformation				4.14	1	0.04
No	179 (89)	182 (95)	1.0			
Yes	21 (11)	18 (5)	6.3 (1.2-31.6)			
Baby’s birth weight				2.82	1	0.09
Above 2500 gms	49 (27)	282 (71)	1.0			
less than 2500 gms	133 (73)	116 (29)	2.4 (0.9-6.7)			
Baby’s Apgar score				26.09	1	<0.001
7-10 at 5 mins	79 (50)	349 (95)	1.0			
0-6 at 5 mins	78 (49)	20 (5)	26.4 (6.1-113.8)			

Reference category for logistic regression represented by 1.0.

Numbers may not add to total sample size due to missing values.

## Discussion

This study examined risk factors associated with fetal and early neonatal mortality at MTRH. Factors that were significantly associated with early neonatal mortality in adjusted analyses were: qualification of the birth attendant; gestational age; number of antenatal visits; maternal complication at birth (PROM, haemorrhage and dystocia); congenital malformations, and low Apgar scores at five minutes.

The odds of low ANC attendance (0-1 visit) were higher for cases relative to controls. This is possibly because fewer ANC visits can result in poorer supervision of the pregnancy and failure to prevent, detect, and manage maternal conditions during the pregnancy. These issues have been reported by other studies in developing countries [19,21]. Moreover, since reasons for low ANC attendance could include lack of education, lack of female empowerment, and poverty, these factors may also explain some of the relationship between ANC attendance and fetal and early neonatal mortality [22]. Additionally, recent research has indicated that timing of visits is more important in detecting complications than the number of visits [23], hence women who only had 0-1 visit may have been prompted by a problem with their pregnancy rather than continuous monitoring of the pregnancy. The timing of ANC was however not captured in our study.

Assistance from a doctor (consultant, registrar or medical officer intern) was protective against neonatal mortality. This finding is in agreement with other studies that have found the presence of a doctor at birth enhances appropriate management and reduces maternal and infant mortality [1,21,24]. Lack of emergency obstetric care increases the risk of neonatal mortality. This is because labouring mothers cannot always access appropriate health services.

Maternal complications that were risk factors for fetal and early neonatal mortality were: PROM, dystocia, and haemorrhage. Mothers with PROM have been previously shown to be more likely to deliver preterm babies than those without this condition, with both maternal and neonatal risk of infection higher for this group [25]. This is concerning as infections are the leading cause of neonatal death in Sub-Saharan Africa [1,10,25]. Dystocia (prolonged or obstructed labour) was also a significant risk factor for mortality and this is consistent with another study which found that dystocia may result in the fetus having asphyxia [26]. Haemorrhage can be rapidly fatal to both the mother and neonate before medical intervention can be instituted and it has been reported as one of the leading causes of neonatal mortality in developing countries [27].

Babies born before or after 37-42 weeks carry significant risks during, and immediately after delivery and may require intervention by doctors [4]. The majority of health facilities in Sub-Saharan Africa lack proper resuscitation equipment and neonatal intensive support units, and are thus unable to adequately manage neonates born prematurely [17,18,28]. Prematurity is among the top three major causes of neonatal death in developing countries because of slow progress in uptake of public health measures such as antenatal corticosteroids, and proper hygiene practices during child birth [3,5,28].

The study found that congenital malformation was significantly associated with mortality. This is consistent with another study that reported congenital malformation as one of the causes of death in developing countries [7]. The presence of congenital anomalies in newborns could be in part explained by the lack of adequate screening and detection of these conditions during the antenatal period. If this had occurred then it may have been possible to give patients and doctors opportunities to make decisions on interventions prior to birth [28-30]. Other pre-disposing factors for congenital anomalies are maternal socioeconomic and nutritional status, the presence of maternal infections, and environmental exposure to hazardous agricultural chemicals which contribute to about one-third of the disease burden in Sub-Saharan Africa [31,32].

The majority of neonatal deaths in this study had poor Apgar scores at five minutes after birth. This is consistent with other studies that have found a high risk of asphyxia among babies born to mothers with poor nutritional status, lack of ANC, and haemorrhage [21,28,33]. Information on some Apgar scores was not recorded, perhaps because of lack of time between transfers from one ward to the other, especially when the neonates had to be rushed to the newborn unit for resuscitation. This is a possible reason for the high proportion of missing values, particularly for the cases, and this may have contributed to the wide confidence intervals for the estimates.

The findings of this study are not only important for MTRH, which is Kenya’s second largest hospital, but also for the Ministry of Health (MOH) in Kenya. They highlight the importance of informing stakeholders about areas where services can be improved. These include, for example, training for newborn care, the provision of adequate supplies and equipment, the development of protocols for newborn management and regular criterion-based audits aimed at averting early neonatal mortality. Additionally, most recommendations from this study relate to education and advocacy, issues which are relevant to the broader community.

One major limitation of this study was that there was potential selection bias because only hospital births were included. The sample may not therefore be representative of all births in the region, because high risk women, or women who develop complications, may be more likely to deliver in hospitals. The proportion of Kenyan women who deliver in community health facilities reported in the literature is 42.8% [34]. Another potential limitation is that it was not possible to blind the data abstractors to case or control status. Also MTRH did not have checklists or protocols for hospital personnel regarding medical records. Time pressures on staff were substantial and there was high patient throughput. The doctor to patient ratio was approximately 1:5000. It is therefore likely that some information may have been omitted from patient records [35].

## Conclusions

In conclusion, the risk factors for fetal and early neonatal mortality included: number of antenatal visits, gestational age, qualification of the birth attendant, mother’s complication at birth (PROM, haemorrhage, dystocia), low Apgar scores at five minutes and congenital malformations. Interventions that focus on educating mothers on the importance of antenatal clinic attendance, as well as ensuring screening, detection, monitoring and management of maternal conditions during the antenatal period, could help reduce neonatal mortality rates.

Doctor attendance at birth and during emergencies is important and can help to ensure that the newborn survives beyond the first week of their life. There is a need to increase the availability of resuscitation equipment, train personnel in newborn care and develop and implement protocols and checklists to promote efficiencies in medical record information gathering and documentation. Accurate complete maternal and neonatal records are important for the delivery of care to both the mother and baby. Combining maternal and neonatal records is one way of assisting clinical management and also providing data for research.

This research offers some possible reasons for the high mortality among neonates in a tertiary institution in Kenya. The findings have relevance for both mothers and neonates. Further research into factors influencing the timing and uptake of antenatal care by women in the community, as well as the contribution of quality of care to neonatal mortality in health facilities, is needed.

## Abbreviations

MTRH: Moi Teaching and Referral Hospital
PROM: Premature rupture of membranes
MDG: Millennium development goals
NBU: Newborn unit
BBA: Born before arrival
ANC: Antenatal care
HIV: Human immunodeficiency virus
RDS: Respiratory distress syndrome
MOH: Ministry of Health
